# A Highly Sensitive Dual-Drive Microfluidic Device for Multiplexed Detection of Respiratory Virus Antigens

**DOI:** 10.3390/mi15060685

**Published:** 2024-05-23

**Authors:** Xiaohui Yang, Yixian Li, Josh Zixi Lee, Yuanmin Sun, Xin Tan, Yijie Liu, Yang Yu, Huiqiang Li, Xue Li

**Affiliations:** 1 Department of Clinical Immunology, School of Medical Laboratory, Tianjin Medical University, Tianjin 300203, China; 16622080665@163.com (X.Y.); liyixian1010@163.com (Y.L.); sunyuanmin1998@163.com (Y.S.); tanxintx0505@163.com (X.T.); yuyangchn@tmu.edu.cn (Y.Y.); lhq@tmu.edu.cn (H.L.); 2 Beijing MicVic Biotech Co., Ltd., Beijing 101200, China; joshzixilee@mic-vic.com (J.Z.L.); liuyijie@mic-vic.com (Y.L.)

**Keywords:** microfluidic, dual-drive, sensitivity, respiratory viruses

## Abstract

Conventional microfluidic systems that rely on capillary force have a fixed structure and limited sensitivity, which cannot meet the demands of clinical applications. Herein, we propose a dual-drive microfluidic device for sensitive and flexible detection of multiple pathogenic microorganisms antigens/antibodies. The device comprises a portable microfluidic analyzer and a dual-drive microfluidic chip. Along with capillary force, a second active driving force is provided by a removable self-driving valve in the waste chamber. The interval between these two driving forces can be adjusted to control the reaction time in the microchannel, optimizing the formation of antigen-antibody complexes and enhancing sensitivity. Moreover, the material used in the self-driving valve can be changed to adjust the active force strength needed for different tests. The device offers quantitative analysis for respiratory syncytial virus antigen and SARS-CoV-2 antigen using a 35 μL sample, delivering results within 5 min. The detection limits of the system were 1.121 ng/mL and 0.447 ng/mL for respiratory syncytial virus recombinant fusion protein and SARS-CoV-2 recombinant nucleoprotein, respectively. Although the dual-drive microfluidic device has been used for immunoassay for respiratory syncytial virus and SARS-CoV-2 in this study, it can be easily adapted to other immunoassay applications by changing the critical reagents.

## 1. Introduction

Point-of-care testing (POCT) enables rapid diagnosis and monitoring of patients in various settings including clinics, airports, schools, and at home, delivering immediate on-site results to the users [1,2,3]. POCT products represent one of the most promising sectors within the in vitro diagnosis (IVD) field [4], playing a vital role in providing diagnoses in relatively resource-poor environments [5,6]. Unlike traditional methods that rely on cumbersome equipment, POCT systems offer the benefits of portability, simplicity, and user-friendliness [7,8]. Furthermore, with rapid advancements in micromotor technology, POCT devices are set to become even more compact, rapid, automated, and intelligent in the future [9].

Colloidal gold immunochromatography assay (GICA) is the most representative POCT method widely used in clinical diagnosis. It has a large market demand due to its rapid detection time, affordable prices, and ease of operation [10]. However, GICA has been known to give a high false-negative rate due to its low sensitivity [5,11]. Additionally, it is challenging to control inter-batch variation, resulting in inaccurate quantitative analysis [12].

Microfluidics is the study of hydrodynamics in micrometer channel dimensions [13], and it has been developed for the miniaturization of chemical tests, offering robust, sensitive, and cost-effective assays [14,15]. These systems are designed to provide a high-throughput, integrated, and automated chemical analysis [16,17,18]. Microfluidics has played an important role in the development of POCTs by offering smaller reagent volume consumption and centralized multiplex sample processing procedures in a single device [17,19]. In addition, microfluidics can be integrated with electrochemical analysis, spectroscopic analysis, chemiluminescent analysis, etc., and have a wide range of applications in many fields, such as biomedical research, synthetic analysis, and forensic identification. Immuno-microfluidics is a new platform for POCT that integrates immunoassay and microfluidic technologies [20]. This new technology platform has brought about breakthroughs in the biomedical field [21,22], integrating the basic operating units of sample processing, separation, reaction, and detection into a single chip, resulting in rapid and efficient detection with low consumption of reagents and samples [23,24].

Capillary force driven microfluidics is a technique for moving fluids in microchannels without expensive or complex internal/external equipment [25]. This type of microfluidic platform is easy to operate, fast, low cost, portable, and allows for multiplexed testing [13,26]. These advantages make it an attractive option for detecting target molecules. The reagents can be pre-coated on the surface of the microchannel, and results obtained from a portable analyzer by loading the patient sample into the microfluidic chip and incubating it for a short time [16]. The simplicity of the detection process led to the development of more streamlined POCT devices, which have been a focal point of research in the scientific community for the past decades.

However, existing capillary force driven microfluidic chips have two main limitations in their sensitivity due to their structural design. Firstly, the antigen-antibody complex formation through non-covalent interactions is a dynamic balance and requires a certain length of reaction time to reach. In addition, the complex may be dissociated under different conditions [27,28,29]. The fluid in the microchannel flows in a laminar flow, leading to insufficient antigen-antibody reaction when the fluid flow is fast. In the design of the current capillary force driven microfluidic chip, the microchannel is directly connected to the waste chamber and the sample fluid does not stay in the microchannel as it moves into the waste chamber directly. This process significantly limits the reaction time of the sample with the different reagents in the microchannel, thus limiting sensitivity. Secondly, because the volume of liquid is limited in a traditional waste chamber, effectively removing the excess signal left in the microchannel remains challenging.

In this context, we present a novel dual-drive microfluidic chip that utilizes both capillary and active forces to control the flow of fluids. It is characterized by a capillary force-driven microchannel paired with a self-driving valve composed of cellulose material in the waste chamber. The self-driving valve bridges the microchannel and the waste chamber by sliding into the sample outlet. First, capillary force is used to move the fluid from the sample chamber into the microchannel; second, an active force provided by the self-driving valve suctions the fluid from the microchannel into the waste chamber. The reaction time can be controlled by adjusting the time interval between the addition of the sample and the triggering of the self-driving valve to provide sufficient time for the formation of the antigen-antibody complex, improving detection sensitivity. Furthermore, background noise can be further eliminated by controlling the velocity and volume of the fluid flow by changing the cellulose material of the self-driving valve. We evaluated the applicability of this novel microfluidic chip with respiratory syncytial virus (RSV) recombinant fusion protein and SARS-CoV-2 recombinant nucleoprotein detection as illustrative examples.

## 2. Materials and Methods

### 2.1. Reagents and Materials

RSV recombinant fusion protein and SARS-CoV-2 recombinant nucleoprotein were purchased from Jiangsu Dongkang Biomedical Technology Co., Ltd. (Nantong, China). Anti-RSV monoclonal antibody (mAb) was purchased from Sino Biological Inc (Beijing, China). Anti-SARS-CoV-2 mAb was purchased from DIA-UP Biotech (Beijing, China). Goat anti-mouse polyclonal antibody was purchased from Applygen (Beijing, China). Streptavidin was purchased from Shanghai Aladdin Biochemical Technology Co., Ltd. (Shanghai, China). Four cellulose materials with different absorbencies were purchased from Nanjing Microdetection Biotechnology Inc. (Nanjing, China). Carboxylate-modified fluorescent microspheres (0.2 μm, dark red) were purchased from Thermo Fisher Scientific (Eugene, OR, USA).

### 2.2. Analytes 

Lysates of viral recombinant proteins were used as analytes for microfluidic chip binding. In order to achieve near-natural analyte conditions, viral recombinant protein was added to healthy donor nasal secretions at specific concentrations.

### 2.3. Conjugation of Fluorescent Microspheres with Antibodies

The fluorescent microspheres were washed with ultrapure water before use. After resuspending the microspheres in the 0.05 M MES buffer (pH 6.2), N-hydroxysuccinimide (NHS) and N-(3-dimethylaminopropyl)-N’-ethylcarbodiimide hydrochloride (EDC) were added to activate the carboxylate-modified microspheres. After incubation on a rotator for 30 min at room temperature, the activated microspheres were washed twice with 0.01 M phosphate buffer (pH 7.4). Then, anti-RSV mAb and SARS-CoV-2 mAb with a final concentration of 0.2 mg/mL were added to the resuspended microspheres and incubated with rotation for 2 h. Subsequently, 2% skim milk powder was added to block the unreacted carboxyl groups, and the reaction mixture was further incubated with rotation for 1 h. After incubation, 1% sodium citrate solution was added to terminate the reaction. The fluorescent microspheres were centrifuged at 15,000 rpm for 20 min to remove the supernatant. After washing twice with phosphate buffer, the fluorescent microspheres were resuspended in a storage buffer (0.01 M phosphate buffer saline with 0.1% Tween-20). Finally, the concentration of the fluorescent microspheres was determined by a UV spectrophotometer (Mettler-Toledo International Inc., Columbus, OH, USA) and stored at 4 °C for subsequent use.

### 2.4. Description of the Microfluidics Analyzer

**Figure 1:**
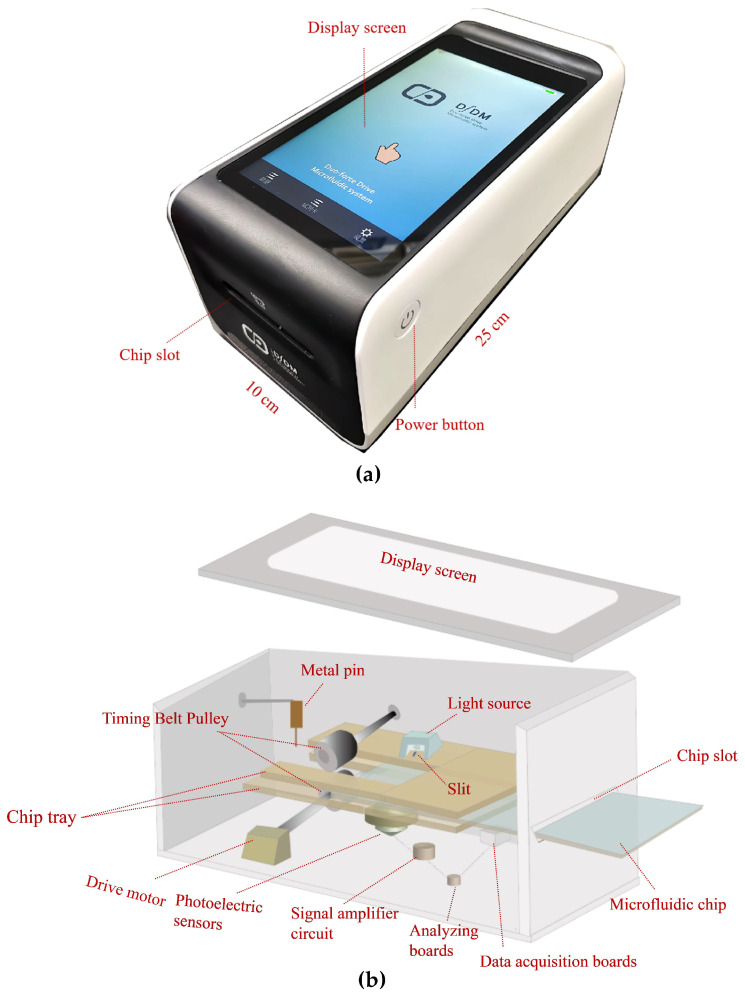
Structure of the microfluidics analyzer. (**a**) Photograph of the microfluidics analyzer. (**b**) Exploded view of the microfluidics analyzer.

Figure 1a,b present a photograph and a schematic illustration of the main structure and components of the microfluidics analyzer. The main elements of the analyzer include a chip tray, a light source, a drive motor, a signal collection system, and a display screen. The operating principle of the analyzer is as follows: first, the microfluidic chip enters the chip tray through the chip slot. Then, the drive motor drives the timing belt pulley and pulls the chip into the chip tray completely. Subsequently, the metal pin is inserted into the circular hole of the self-driving valve located in the waste chamber via the stepper motor control. After the pin is secure, the chip tray is moved to push the self-driving valve to slide by pressing it into the pin. The resulting movement can control how the self-driving valve is embedded/detached from the end of the microchannel, thus connecting/disconnecting the microchannel to the waste chamber. Subsequently, the light source generates a laser beam at 680 nm, which is irradiated through the slit onto the microchannel of the chip. Afterwards, the photoelectric sensor receives the fluorescence generated from the fluorescent microspheres. The photoelectric sensor output is equipped with a signal amplifier circuit, which can amplify the signal output from the sensor and then pass it to the analyzing boards. Ultimately, the fluorescence signal is measured and results are fed back on the display screen.

### 2.5. Dual-Drive Microfluidic Chip Design and Assembly

**Figure 2:**
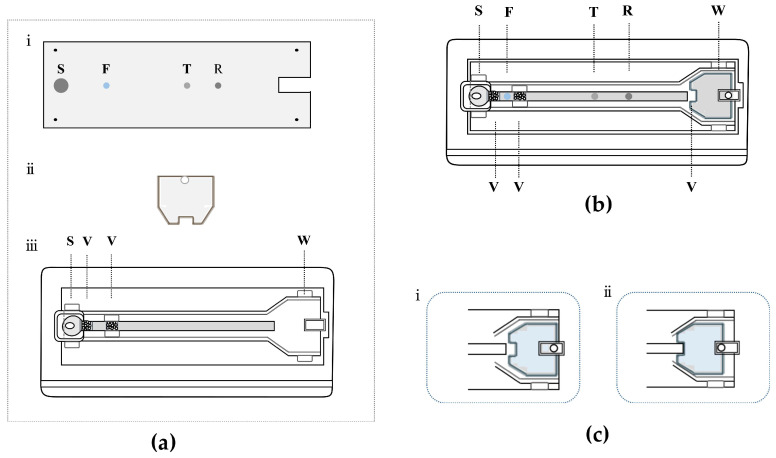
Schematic diagram of the dual-drive microfluidic chip. (**a**) The exploded view of the dual-drive microfluidic chip: (i) PMMA substrate layer; (ii) Self-driving valve layer. (iii) PMMA cover layer. (**b**) The overall view of the assembled dual-drive microfluidic chip. (**c**) The detailed arrangement of the waste chamber: (i) the self-driving valve is away from the microchannel, and the microchannel is unconnected to the waste chamber; (ii) the self-driving valve is embedded in the microchannel, and the microchannel is connected to the waste chamber; the cellulose material (marked in blue) of the self-driving valve can be replaced. S, sample region; F, fluorescence region; T, test region; R, reference region; W, waste chamber; V, valve.

Figure 2a,b show an exploded view and an overall view of the proposed dual-drive microfluidic chip. As shown in Figure 2a, the dual-drive microfluidic chip consists of a PMMA substrate layer, a self-driving valve layer, and a PMMA cover layer. The PMMA substrate layer (25 mm wide, 75 mm long, and 1 mm high) contains four functional regions: sample region (S), fluorescence region (F), test region (T), and reference region (R). Biomolecules are immobilized in the PMMA substrate layer, and the procedure is described in the next section. The main channel structure is arranged in PMMA cover layer (40 mm wide, 85 mm long, and 2 mm high), which is patterned with a rectangular sample chamber (5 mm wide, 8 mm long, and 3 mm high) and a waste chamber (2 mm deep) connected by a microchannel. In addition, the microchannel contains two flow control valves, which are placed on the sides of the fluorescence region. The self-driving valve consists of a cellulose material with a thickness of 1 mm, shaped to match the dimensions of the waste chamber, and is placed inside the waste chamber during assembly. In addition, one side of the self-driving valve has a 1.5 mm diameter circular hole that allows a metal pin to be inserted, allowing the self-driving valve to be moved by the horizontal push of the metal pin. The self-driving valve can be replaced with different types or thicknesses of cellulose materials according to the demands of different tests. The above structures were designed using AutoCAD software, Version 2021 (Autodesk, San Francisco, CA, USA) and fabricated using a CO_2_ laser etcher (Visutec, Brazil). 

The assembly procedure of the dual-drive microfluidic chip is as follows: first, the self-driving valve material is placed into the waste chamber of the PMMA cover layer. Then, the PMMA substrate layer with immobilized biomolecules is tightly affixed to the PMMA cover layer, and a reaction microchannel (3 mm wide, 60 mm long, and 20 μm high) is formed by the gap between two layers and then adhesively bonded with acetone. Finally, the chip is dried and stored at room temperature for further use.

### 2.6. Immobilization of Biomolecules on the PMMA Substrate Layer

First, 1.5 μL of activated streptavidin is spotted in the test region and reference region, respectively. The chip is subsequently incubated in a closed incubator (Shanghai Boxun Medical Biological Instrument Corp, Shanghai, China) for 1 h at constant humidity and temperature. Then, the chip is washed with TRIS solution. After drying, 1.6 μL of biotinylated antibody solution is spotted in both the test region and reference region and incubated for an additional 1 h. The unbound sites are blocked with 1% bovine serum albumin (BSA) to prevent non-specific interactions. The chip is then washed and dried in a similar way as described above. Finally, 1.2 μL of antibody-coupled fluorescent microspheres solution is spotted in the fluorescent region, and 10 μL of sample buffer is spotted in the sample region. The chip is then placed in an electrothermal heated drying cabinet (Shanghai Lichen Instrument Inc., Shanghai, China) at 37 °C for 40 min. 

### 2.7. Working Principle of Dual-Drive Microfluidic Chip

**Figure 3:**
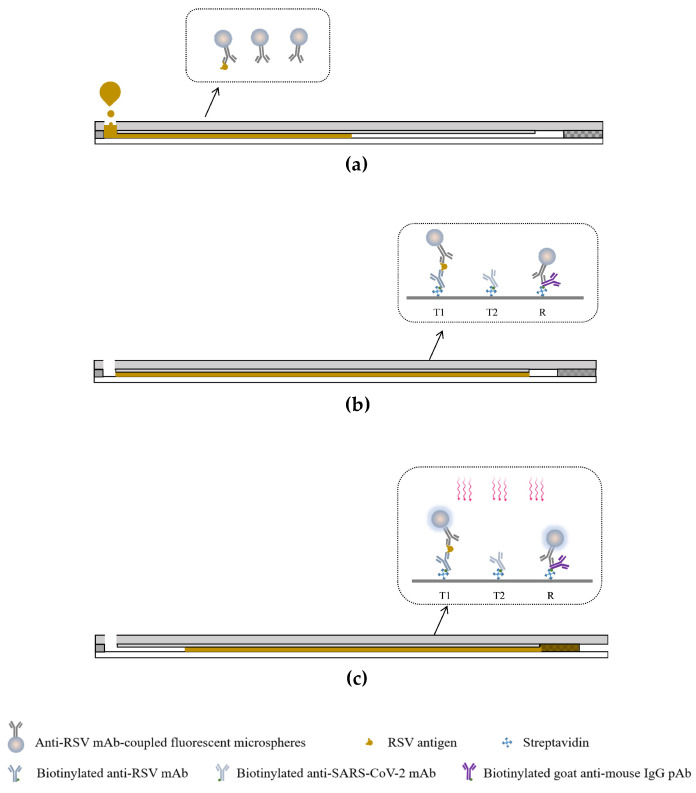
Working principle of dual-drive microfluidic chip. (**a**) Add samples to the sample chamber. (**b**) Reaction phase. (**c**) The analyzer excites and reads the fluorescence signals in the detection and reference regions.

Figure 3 illustrates the principle for viral antigen detection. Initially, the self-driving valve in the waste chamber is detached from the sample outlet, which keeps the microchannel disconnected from the waste chamber. Subsequently, 35 μL of the sample was loaded into the sample chamber and moved into the microchannel by capillary force. The sample dissolves the spotted antibody-coupled fluorescent microspheres and is retained in the microchannel for further reaction. The viral antigen in the sample initially combines with the antibody-coupled fluorescent microspheres, and the resulting complex is captured by the precoated antibody in the test region. Unbound fluorescent microspheres are captured by the precoated biotinylated goat anti-mouse antibody in the reference region. After 90 s, the self-driving valve in the waste chamber is embedded into the sample outlet connecting the microchannel with the waste chamber. The sample fluid in the microchannel is then slowly moved into the waste chamber. During this process, target molecules in the sample are captured by the antibody in the test region, while excess sample fluid is washed away and collected into the waste chamber. Finally, the analyzer measures the fluorescence signals in the microchannel at 680 nm automatically. For subsequent detection of RSV and SARS-CoV-2 antigens, we fabricated a microfluidic chip that contains two test regions, where the first test region (T1) is used for RSV antigen detection, and the second test region (T2) is used for SARS-CoV-2 antigen detection.

### 2.8. Validation and Optimization of the Dual-Drive Microfluidic System

The performance of the microfluidic analyzer was verified using a calibration chip consisting of varying fluorescence intensity. This is achieved by immobilizing different quantities of fluorescent microspheres covering the entire measurement range of the optoelectrical biosensor of the analyzer within the test region. The calibration chip was scanned 10 times in an analytical run to evaluate the precision of the analyzer. A calibration chip with more point arrays was used to evaluate the measuring range of the analyzer. 

The reaction time in the microchannel can be controlled to potentially increase sensitivity. To test this, we compared the two models to validate the value of dual-drive microfluidic chips in improving sensitivity. Model 1 is to initially attach the self-driving valve to the sample outlet, connecting the microchannel to the waste chamber. When the sample enters the microchannel, it flows into the waste chamber directly without significant retention time. After the reaction was complete, the signal values of the T and R regions were measured by the analyzer. Conversely, model 2 starts with the self-driving valve detached from the sample outlet, disconnecting the microchannel from the waste chamber. The sample flows into the microchannel when added and is retained in the microchannel for two minutes. Afterwards, the self-driving valve was moved to attach the sample outlet, moving the sample from the microchannel to the waste chamber. Subsequently, T and R region signal values were measured by the analyzer. Finally, the T/R ratios for both models were calculated and compared against each other.

Along with different models of detection, the sensitivity of the dual-drive microfluidic system can also be influenced by several factors, including the cellulose material used in the self-driving valve, and the length of sample reaction time before being moved into the waste chamber. To further elucidate the impact of these conditions, we first tested 4 different kinds of cellulose materials using RSV and SARS-CoV-2 antigen-positive samples (both prepared from recombinant antigen), with nasopharyngeal samples from the individuals without infection as negative controls. Second, we tested the optimal reaction incubation time by using varying concentrations of RSV and SARS-CoV-2 antigen samples. The resulting T and R region signal values were measured for each experiment, and the T/R ratios were analyzed to deduce the optimal cellulose material and incubation time for the dual-drive microfluidic system. 

## 3. Results

### 3.1. Performance of the Microfluidics Analyzer

The precision of the microfluidic analyzer was evaluated by scanning a calibration chip, which contains an array of samples with different fluorescence intensities. The calibration chip was measured 10 times, with the mean, standard deviation (SD), and relative standard deviation (RSD) calculated. As shown in Table 1, the RSD ranged from 0.003% to 1.180%, suggesting excellent precision of the instrument.

**Figure 4:**
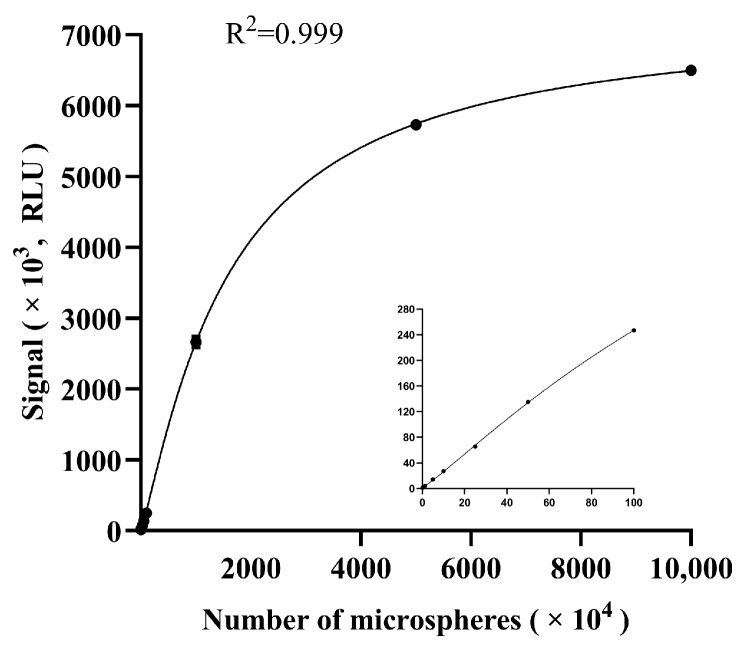
The measurement range of the analyzer. The number of microspheres were 0.16 × 10^4^, 1.25 × 10^4^, 5 × 10^4^, 10 × 10^4^, 25 × 10^4^, 50 × 10^4^, 100 × 10^4^, 1000 × 10^4^, 5000 × 10^4^ and 10,000 × 10^4^ (from left to right), and the embedded small graph shows the zoomed-in graphs for the numbers of microspheres 0.16 × 10^4^ to 100 × 10^4^. The error bars show standard deviations of triplicate measurements.

Similarly, to evaluate the measurement range of the microfluidic analyzer, 10 samples with different numbers of fluorescent microspheres were arranged on the calibration chip. Afterwards, each sample was scanned 3 times and the mean signal value was calculated. The results showed that the measurement range of the instrument was 0 RLU-6,500,000 RLU (Figure 4), which covered a wide range of most samples and was considered acceptable in clinical practice.

### 3.2. Validating the Value of the Dual-Drive Microfluidic Chip in Improving Sensitivity

**Figure 5:**
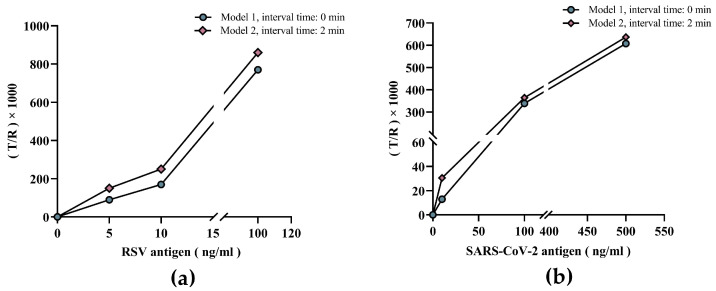
Validation of dual-drive microfluidic chip for improved sensitivity. (**a**) Comparison of RSV control samples with different concentrations in the two models. (**b**) Comparison of SARS-CoV-2 control samples with different concentrations in the two models.

Compared to Model 1, the T/R values of low-concentration RSV samples were significantly higher in Model 2 (Figure 5a). This was further validated by three samples with different concentrations of SARS-CoV-2 antigen with similar conclusions to the RSV results (Figure 5b). In other words, increasing the reaction time by the dual-drive microfluidic chip improved detection sensitivity, which further illustrates the value and effectiveness of the chip design in allowing the control of incubation time length.

### 3.3. System Optimization of Dual-Drive Microfluidic Chip

**Figure 6:**
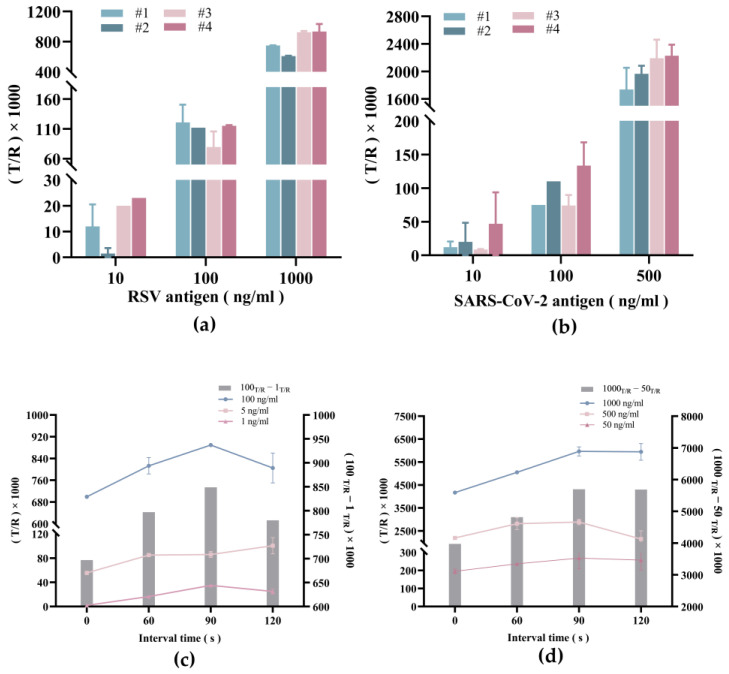
Optimization of parameters for RSV and SARS-CoV-2 antigen assays. (**a**) Effect of cellulose material on the results of RSV control samples with different concentrations. (**b**) Effect of cellulose material on the results of SARS-CoV-2 control samples with different concentrations. (**c**) Effect of incubation time on the results of RSV control samples with different concentrations. (**d**) Effect of incubation time on the results of SARS-CoV-2 control samples with different concentrations.

Figure 6a shows the T/R values of the RSV control samples with concentrations of 10 ng/mL, 100 ng/mL, and 1000 ng/mL obtained from four different chips. In both cases, the T/R value was highest when using cellulose material #4. In addition, similar results were obtained when detecting the SARS-CoV-2 sample (Figure 6b). Therefore, we surmise that cellulose material #4 is a superior material for the self-driving valve.

Next, the optimal reaction incubation time was determined by measuring sensitivity and the measurement range along different concentrations of RSV and SARS-Cov-2 antigen. As shown in Figure 6c,d, the T/R value peaked when the incubation time was increased to 90 s but then showed marginal reduction as the time length was further increased to 120 s. This suggests that the time for the antigen-antibody reaction to reach a dynamic balance is around 90 s and represents the optimal incubation time length before triggering the self-driving valve.

### 3.4. Detection Capability of Respiratory Virus Antigens by Dual-Drive Microfluidic Chip

**Figure 7:**
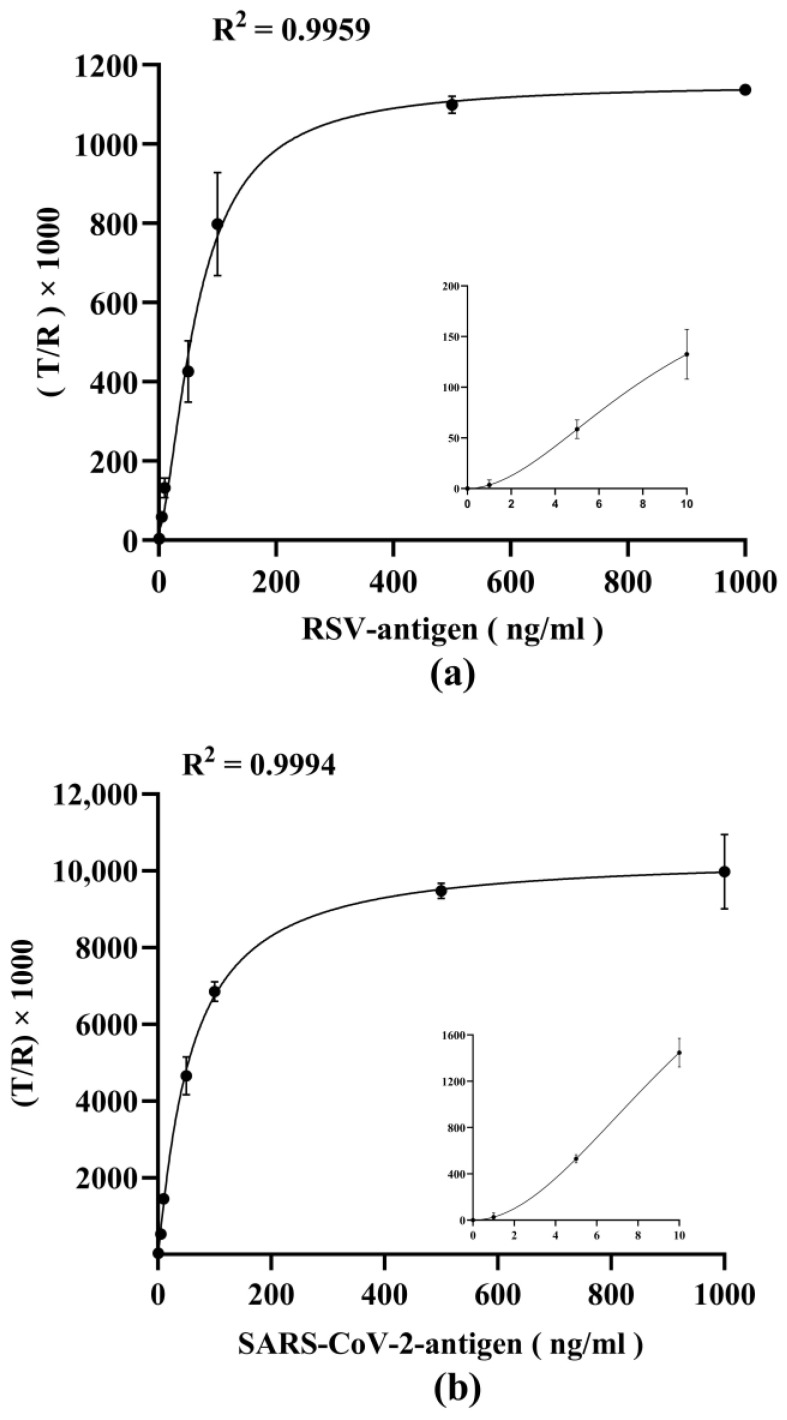
Establishment of the calibration curve. (**a**) Calibration curve of dual-drive microfluidic chip for detecting RSV antigen based on four-parameter logistic equation. (**b**) Calibration curve of dual-drive microfluidic chip for detecting SARS-CoV-2 antigen based on four-parameter logistic equation.

Since a quantitative micro-immunoassay should provide results in units related to a standard, calibration curves for antigen concentration determination were performed by measuring serial dilutions (from 0 ng/mL to 1000 ng/mL in viral lysate) of viral antigens. As shown in Figure 7a,b, the trend fits well in four-parameter logistic curves, with a R^2^ > 0.995. The curve equations are y = 1126.35/[1 + (x/66.23) − 1.58] + 25.65 (for RSV antigen detection), and y = 10433.32/[1 + (x/57.12) − 1.10] − 41.88 (for SARS-CoV-2 antigen detection), respectively. The limits of detection (LoD) and limit of quantification (LoQ) are shown in Table 2. The LoD values were conservatively calculated by using the average value of 10 blank samples plus 3 times the standard deviation, and the LoD for RSV antigen and SARS-CoV-2 antigen were calculated to be 1.121 ng/mL, and 0.447 ng/mL, respectively. The LoQ values were determined by measuring the 10 blank samples and calculating the mean of the signals plus 10 times the standard deviation. The LoQ for RSV antigen and SARS-CoV-2 antigen were calculated to be 2.717 ng/mL, and 0.580 ng/mL, respectively. This shows roust sensitivity in the detection of viral antigens via the dual-force microfluidic system.

## 4. Discussion

Early identification of pathogenic agents is of great importance in monitoring and controlling infectious disease epidemics [30,31,32], especially since the impact of the COVID-19 pandemic has reinforced the need for rapid, sensitive, reliable, and cost-effective POCT devices for large-scale population screening [33,34]. Compared with traditional detection platforms, microfluidic technology integrates multiple reaction processes into a single microchannel, which has the advantages of lower reagent usage, streamlined operation, and improved portability while at reduced cost [20,35,36]. Multiple microfluidic sensors have undergone field testing and show great potential for POCT applications [37], bringing new opportunities for efficient detection of pathogenic agents. 

Controlling the flow of fluid is a crucial part of a microfluidic chip, and is typically achieved by utilizing two types of chips, active or passive, depending on the force driving the fluid [24]. The active microfluidic chip requires additional power sources, such as pressure, centrifugal force, electroosmotic force, etc. The instrument’s internal mechanical components precisely control the liquid flow in the microchannel, allowing real-time monitoring of the liquid flow within the chip [38]. However, active microfluidic chips have unavoidable shortcomings. Firstly, the need for external force generation devices increases the complexity of the instrument and chip design, which increases the overall cost of the assay. Secondly, its more complex features often require professional training for operation and are not suitable for ordinary users [39]. These intrinsic shortcomings limit the application and development of active microfluidic chips in the field of rapid detection. On the other hand, passive microfluidic chips drive the fluid in the microchannel via surface hydrophilicity or capillary force, instead of utilizing an additional power source [40]. The path, flow rate, and state of the fluid in the microchannel can be controlled by adjusting the microchannel structure, changing the substrate material, or modifying the hydrophobicity of the chip surface [41]. For example, Tu et al. propose a microfluidic paper-based analysis device with a localized dissolvable delay by installing a dissolvable delay region behind the test and reference lines, which effectively reduces the flow rate and ensures sufficient sensitivity in the assay [42]. Li et al. demonstrate a dual three-dimensional microfluidic paper-based device with branched 3D microfluidic channels to obtain solution migration time delays from one detection region to another [43].

In this study, we developed a dual-drive microfluidic device, which comprises a portable analyzer (18 cm long, 9 cm wide, and 8 cm high) and a dual-drive microfluidic chip that combines the advantages of both active and passive microfluidic chip systems. The analyzer facilitates assay operation by non-professional users through a user-friendly and intuitive interface that guides users through the testing process. Furthermore, the analyzer demonstrates excellent precision and a wide signal detection range. The dual-drive microfluidic chip is comprised of five regions arranged along the sample flow direction: sample region, fluorescence region, test region, reference region, and waste chamber. Two passive valves are located on each side of the fluorescence region, the first valve functions to mix the sample with the pre-dried sample buffer spotted in the sample region and provide the appropriate conditions for the subsequent reaction; the second valve is used to allow the sample to fully react with the fluorescent microspheres-coupled antibody in the fluorescence region. Since each microfluidic chip is produced and operated independently, a reference region is necessary to evaluate the effectiveness of the chips, and to normalize the final results to eliminate small variations in fluid dynamics, ensuring good detection accuracy.

The reaction time for the antigen-antibody complex to reach a dynamic balance can vary highly between different antibody-antigen pairs. However, current microfluidic chips driven by passive forces (e.g., capillary force) are unable to dynamically control the reaction time in the microchannel tailored to each reaction, making it difficult to meet the different demands of different tests. Given this limitation, the highlight of our system is the innovation of a self-driving valve (composed of cellulose material) in the chip waste chamber, which can be moved to connect the microchannel to the waste chamber. To illustrate the process in more detail: the sample added flows into the microchannel driven by capillary force and remains at the end of the microchannel due to surface tension. The sample will be retained in the microchannel as the self-driving valve is detached from the microchannel, allowing the antigen/antibody in the sample to react with the pre-immobilized antibody/antigen in the microchannel. Once the antigen/antibody in the sample and the pre-immobilized antibody/antigen in the microchannel have sufficiently reacted with each other, the self-driving valve is triggered. It is moved to embed into the sample outlet connecting the microchannel to the waste chamber, allowing the excess liquid to wash through the microchannel, and collected in the waste chamber. Our research suggests that appropriately extending the incubation time is beneficial for enhancing sensitivity. Determination of the best duration for multiplexed assays requires a balance of different factors, such as non-specific binding, or the impact on assay throughput. Since the ideal reaction times for various detection targets are different, when integrating them into a single chip, the ideal reaction time is selected based on the item with the highest sensitivity requirements.

Existing passive (e.g., capillary force) microfluidic chips often exhibit a high background signal, which can mask low-concentration samples and lead to false-negative results in multiplexed tests. It is caused by the limited fluid capacity of the waste chamber, causing excessive signal (e.g., fluorescence microspheres) left in the microchannel. In our proposed dual-drive microfluidic chip, the waste chamber can accommodate increasing volume of fluid by adjusting the cellulose material used in the self-driving valve, as well as tailoring the flow rate to fit the needs of different reactions.

In our research findings, for the detection of RSV and SARS CoV-2 antigen via the dual-drive microfluidic system, the LoD was 1.212 ng/mL and 0.447 ng/mL, respectively, and the LoQ was 2.717 ng/mL and 0.580 ng/mL, respectively. LoD and LoQ were lower than those reported in previously published articles [33], suggesting that the dual-drive microfluidic chip has a greater sensitivity than existing technologies. Further studies are planned to validate the developed dual-drive microfluidic chip using nasopharyngeal swab samples obtained from patients. In addition, more virus species (e.g., influenza virus, adenovirus, norovirus, rotavirus, etc.) or other target analytes (e.g., alpha-fetoprotein, carcinoembryonic antigen, cardiac troponin, etc.) will be considered for multiplex analysis to establish a higher throughput detection platform.

## 5. Conclusions

This work presents a highly sensitive dual-drive microfluidic device as a novel POCT analytical platform with the potential to detect multiple pathogenic microorganisms antigens/antibodies in clinical and non-clinical settings. This platform is cost-effective, making it affordable to a wide range of clinical settings, especially primary healthcare centers, particularly in remote or underdeveloped regions with inadequate medical infrastructure and resources. Additionally, the flexibility of this analytical platform allows for expansion into the clinical diagnosis of other viruses, microorganisms, biomarkers, and other target analytes on demand, providing significant benefits to the healthcare system.

## Figures and Tables

**Table 1:** Precision of the analyzer.

Number of Beads	Mean (RLU)	SD (RLU)	RSD (%)
5 × 10^7^	6,501,661	170	0.003
1 × 10^7^	3,797,041	44,224	1.165
2 × 10^6^	798,692	9422	1.180
4 × 10^5^	157,040	1745	1.111

SD, standard deviation; RSD, relative standard deviation.

**Table 2:** Analytical sensitivity.

Detection Items	LoD	LoQ
RSV	1.121	2.717
SARS-CoV-2	0.447	0.580

LoD, limit of detection; LoQ, limit of quantification; All values are expressed in ng/mL.

## Data Availability

The data presented in this study are available upon request from the corresponding author.
